# Supplementary material for: NMR Evidence for the Topologically Nontrivial Nature in a Family of Half-Heusler Compounds
Source: Sci Rep. 2016 Mar 16;6:23172. doi: 10.1038/srep23172 (PMC4793261; doi:10.1038/srep23172)
Supplement: Supplementary Information [file srep23172-s1.pdf]

Supplemental Materials

NMR Evidence for the Topologically Nontrivial Nature in a Family of Half-Heusler Compounds

Xiaoming Zhang, Zhipeng Hou, Yue Wang , Guizhou Xu, Chenglong Shi, EnKe Liu, Xuekui Xi*, Wenhong Wang*, Guangheng Wu, and Xi-xiang Zhang

*Correspondence and requests for materials should be addressed to X.K.X (email: xi@iphy.ac.cn) or W.H.W (email: wenhong.wang@iphy.ac.cn).

Contents:

I. Experimental and computational details;

(i) Sample preparation;

(ii) Materials characterization;

(iii) Band structure and NMR computations;

II. Experimental results on structural characterizations and NMR;

(i) Structural characterizations for half-Heusler R-M-Bi alloys;

(ii) NMR measurements at different temperatures for R-M-Bi alloys;

III. Computational results on band structure and NMR;

(i) NMR calculations for half-Heusler R-M-Bi alloys;

(ii) Calculations for $Cd_{1-x}Hg_xTe$ (x=0, 0.25, 0.5, 0.75, 1) system;

Supplemental reference;

Supplemental Figure S1-S5;

I. Experimental and computational details

(i) Sample preparation

Single crystals of half-Heusler R-M-Bi (R= Sc, Y, Lu, M= Ni, Pd, Pt) were grown using the self-flux method and constituents were placed in a high-purity argon atmosphere. Proper ratios of high-purity metals (>99.95%) were used as starting materials, which were mixed with fluxed Bi powders in an atomic ratio of 1:10, placed in a tantalum crucible and then loaded into a fused quartz tube. To estimate the best ratio of the fluxed material to RMBi, we examined the mixtures with various weight ratios, ranging from 20:1 to 1:1. The 10:1 mixture resulted in the best solubility and thus, crystal growth. The tube was sealed with Ar gas under a pressure of 1024 Torr and then placed in a furnace. We also tried to estimate the best cooling rate. The best growth conditions at the 10:1 ratio were as follows: the mixed sample was heated from room temperature to 1150℃ for 24 h, maintained at 1150℃ for 24 h and finally slowly cooled to 850℃ at a rate of 2℃/hour.

Single crystals of half-Heusler R-M-Bi (R= Sc, Y, Lu, M= Ni, Pd, Pt) were grown by using the self-flux method. The high-purity starting materials Sc, Y, Lu (ingot, 99.99%), Ni, Pd, Pt (ingot, 99.99%) and Bi (ingot, 99.99%) were mixed together in a molar ratio of 1:1:10, and afterward the mixture was placed in an alumina crucible. This process was performed in a glove box filled with Ar gas, where the oxygen and humidity content was less than 0.5 ppm. The whole assembly was sealed inside a tantalum tube with Ar gas and sealed into an evacuated quartz tube. Crystal growth took place in a furnace by heating the tube from room temperature up to 1150℃ over a period of 15 h, and maintained for 24 h before slowly cooling to 650℃ at a rate of 2℃/h. The

excess Bi flux was removed by spinning the tube in a centrifuge at 650℃. After the centrifugation process, most of the flux contamination was removed from crystal surfaces and the remaining topical flux was etched by diluted hydrochloric acid. Fig. S1 (a)-(d) show the scanning electron microscopic images of six typical single crystals (ScNiBi, ScPdBi, YNiBi, YPdBi, LuPdBi, LuPtBi, $LuPd_{0.1}Pt_{0.9}Bi$ and $LuPd_{0.8}Pt_{0.2}Bi$) after removing excess Bi fluxes as are listed in (b), where apparently well developed (111) planes can be observed.

(ii) Materials characterization;

The composition of the single-crystal samples was determined by energy-dispersive X-ray (EDX) spectroscopy. The crystal structure was then checked by powder X-ray diffraction (XRD) measurement, which was performed on crushed single crystals using a Rigaku X-ray diffractometer with Cu- Kα radiation. The single-crystal orientation was checked by a standard Laue diffraction technique.

(iii) Band structure and NMR computations;

Band structure calculations were performed using the full-potential linear-augmented plane wave code implemented in the WIEN2K package [1, 2]. The converged ground state was obtained using 10 000 k points in the first Brillouin zone. A combination of a modified Becke-Johnson exchange potential and the correlation potential of the local-density approximation were used to obtain the band structures [3], as it predicts band gaps and the band order with favorable accuracy. Spin-orbit coupling (SOC) was treated as a second variational procedure with scalar-relativistic orbitals as a basis, where states up to 10 Ry above the Fermi level were included in the basis expansion.

Chemical shielding and EFG tensors were calculated using density functional theory (DFT), as implemented in NMR- WIEN2K [4], where both cases for scalar and fully relativistic effective core potentials were considered.

II. Experimental results on structural characterizations

(i) Structural characterizations for half-Heusler R-M-Bi alloys

Fig. S1 (a) illustrates that R-M-Bi alloys crystallize into a cubic MgAgAs structure with space group number 216 (F-43m). Rare earth metal (R), Bi and transition metal (M) atoms occupied positions 4a (0, 0, 0), 4b (1/2, 1/2, 1/2) and 4c (1/4, 1/4, 1/4), respectively. The composition of the single-crystal samples was determined by EDX spectroscopy, which was equipped on a Hitachi S-4800 SEM. The EDX measurements were performed at different positions on a crystal surface within an instrument accuracy of 1-2%, and the analysis was performed at numbered points on the crystal surface; for example, see ScPdBi and LuPtBi in Fig. S1 (c). The average R : M : Bi ratio is 1 : 1.04 : 1.07 for ScPdBi, and 1 : 1.03 : 1.09 for LuPtBi, respectively, which are both nearly equal to the molar ratio 1 : 1 : 1. The crystal structures of the single crystals were further checked by XRD measurement. The power XRD measurements of pulverized R-T-Bi crystals showed very sharp and strong peaks and all of them can be indexed to a MgAgAs-type structure, indicating a pure phase of the materials. Here Fig. S2 we only listed the results for LuPdBi and YPdBi, and the refined lattice parameters are 6.57 and 6.63 Å, respectively.

All these results suggest the high quality of our single crystals. To be noted,

we have only listed only part of our results in Fig. S1 and S2 for clarity purpose, and some dates are taken from previous works [5-8].

(ii) NMR measurements at different temperatures for R-M-Bi alloys;

Here, we have shown that a measured ^{209}Bi shift correlates with SOC strength E_{SOC} and ^{209}Bi chemical shielding, calculated from band structure. Although there has been some success performing calculations using experimental lattice constants to describe situations that are difficult to illustrate with experimental results, band structure actually reflects the electronic character of 0K. Therefore, for higher integrity results, here, we compared the ^{209}Bi shift of R-M-Bi alloys at room temperature (the values used in context) at 0K (fitted ones). Fig. S3 shows the temperature dependence of the ^{209}Bi shift for half-Heusler R-M-Bi alloys. For semiconductors or semimetals, the following equation is believed to well work [9, 10]:

$$K = K_0 + C\sqrt{T}e^{-\Delta\varepsilon/k_B T} \qquad \text{Eq. (1)}$$

Where K_0 is a combination of orbital- and temperature-independent shifts, C is a constant,$\Delta\varepsilon$ is the excitation energy and k_B is the Boltzmann constant. The change of temperature will affect the thermally activated density of carriers, which further influences the NMR shifts K. The black dashed curves in Fig. S2 represent fits to Eq. (1). From the inset of Fig. S3, the difference of ^{209}Bi shifts between 300K and 0K were very small (less than 100ppm), without considering LuPtBi [11]. Therefore, in this context the values of ^{209}Bi shifts at room temperature (~300K) used here were appropriate for our work. The reason why ^{209}Bi shift in LuPtBi shows strong temperature dependence may relate to the electron- and hole-pockets as present in the band structure [7].

III. Computational results on band structure and NMR

(i) NMR calculations for half-Heusler R-M-Bi alloys

Fig. S4 compares the NMR results with and without SOC calculations of half-Heusler R-M-Bi alloys. Calculations of NMR chemical shielding are based on DFT, which has been very successful for many materials [4, 12-14]. Usually, calculated values need to be fit linearly to experimental values because of the different reference criterions [4, 12-14]. In Fig. S4, the calculated ^{209}Bi chemical shielding σ_{iso} values without (hollow circles with plus) and with (solid circles) consideration for SOC for R-M-Bi are both shown versus experimentally measured ^{209}Bi shifts. It is clear that, with respect to SOC, the calculated shifts nearly follow linearly with the measured shifts, while without SOC we observe the opposite. This difference indicates that SOC is critical to NMR hyperfine shifts in this alloy system, which is consistent with our other experimental results and the relativistic $p_{1/2}$ shielding via Fermi-contact-like mechanism described in the context of this paper.

(ii) Calculations for the $Cd_{1-x}Hg_xTe$ (x=0, 0.25, 0.5, 0.75, 1) system

To further support our view that SOC scales with NMR isotropic shifts in heavy-atom containing system in solids, here, we discuss $Cd_{1-x}Hg_xTe$ (x=0, 0.25, 0.5, 0.75, 1), a well-studied system in which and our relativistic $p_{1/2}$ shielding view should fit perfectly.

When x=0 and x=1, the crystal lattice structures of pure CdTe and HgTe are

zinc blend (space group F-43m, 216) with lattice constants 6.48 and 6.46 Å, respectively. Fig. S5 (a) shows the crystal structure. By assigning the number of atoms of Cd:Te as 3:1, 1:1 and 1:3 in the unit cell, we built the structure for x=0.25, 0.5 and 0.75, and we artificially set the lattice constant as 6.46 Å (the average of CdTe and HgTe). Fig. S5 (b) shows electronic band structures with SOC for $Cd_{1-x}Hg_xTe$. The bands with Γ_8 and Γ_7 symmetries are denoted in blue and red, respectively. The details of the bands for CdTe and HgTe were consistent with previous work [15]. Fig. S5 (c) shows the measured and calculated NMR ^{125}Te isotropic shifts, and the calculated SOC parameter E_{SOC} versus Hg concentration. We provide only the reliable values of experimental ^{125}Te shifts for pure CdTe and HgTe, while those for other Hg concentrations were difficult to trace because of the occurrence of massive clusters [16]. Shifts calculated with SOC fit with the experimental values much better than those calculated without SOC, indicating that SOC played an important role in NMR shifts. With increased Hg concentration, the calculated SOC strength (absolute value of E_{SOC}) decreased, although the Hg atom is much heavier than the Cd atom, which is ascribed to a lack of inversion symmetry in the zinc-blende structure [15, 17]. Interestingly, by setting both scales of ^{125}Te shifts and calculated E_{SOC} proportional to those of the $LuPd_{1-x}Pt_xBi$ system, the calculated ^{125}Te shifts and E_{SOC} nearly became parallel upon Hg concentration (see pink lines). The same response was also evident in the $Cd_{1-x}Hg_xTe$ (x=0, 0.25, 0.5, 0.75, 1) system, such that the NMR shifts scaled with SOC strength,

although the difference of ^{125}Te shifts between CdTe and HgTe were not as strong as that in the half-Heusler R-M-Bi system.

SUPPLEMENTARY REFERENCES

[1]. D. J. Singh, Plane Waves, Pseudopotentials and the LAPW Method (Kluwer Academic, Boston, 1994).

[2]. P. Blaha, K. Schwarz, G. Madsen, D. Kvaniscka, and J. Luitz, Wien2k, An Augmented Plane Wave Plus Local Orbitals Program for Calculating Crystal Properties (Vienna University of Technology, Vienna, Austria, 2001).

[3]. F. Tran and P. Blaha, Phys. Rev. Lett. 102, 226401 (2009).

[4]. R. Laskowski and Peter Blaha, Phys. Rev. B 85, 035132 (2012).

[5]. W. H. Wang, Y. Du, G. Z. Xu, X. M. Zhang, E. K. Liu, Z. Y. Liu, Y. G. Shi, J. L. Chen, G. H. Wu, and X.-X. Zhang, Sci. Rep. 3, 2181 (2013).

[6]. G. Z. Xu, W. H. Wang, X. M. Zhang, Y. Du, E. K. Liu, S. g. Wang, G. H.Wu, Z. Y. Liu, and X. X. Zhang. Sci. Rep. 4, 5709 (2014).

[7]. Z. P. Hou, G. Z. Xu, X. M. Zhang，Z .Y. Wei, S. P. Shen, E. K. Liu, Y. S. Chai, Y. Sun, X. K. Xi, Z. Y. Liu, W. H. Wang, G. H. Wu and X. X. Zhang. “Extremely high electron mobility and large magnetoresistance in a half-Heusler semimetal LuPtBi”, unpublished.

[8]. Z. P. Hou, G. Z. Xu, X. M. Zhang，Z .Y. Wei, E. K. Liu, X. K. Xi, Z. Y. Liu, W. H. Wang, G. H. Wu and X. X. Zhang. “synthesis and transport properties of half-Heusler single crystal ScPtBi”, unpublished.

[9]. N. Bloembergen, Physica (Amsterdam) 20, 1130 (1954); Dieter Wolf, Spin Temperature and Nuclear-spin Relaxation in Matter (Clarendon, Oxford, 1979).

[10]. C. S. Lue and Joseph H. Ross, Phys. Rev. B 63, 054420 (2001).

[11]. B. Nowak, O. Pavlosiuk, and D. Kaczorowski, J. Phys. Chem. C 119, 2770 (2015).

[12]. R. Laskowski and Peter Blaha, Phys. Rev. B 89, 014402 (2014).

[13]. R. Laskowski and Peter Blaha, J. Phys. Chem. C 119, 731 (2015).

[14]. A. Sadoc, M. Body, C. Legein, M. Biswal, F. Fayon, X. Rocquefeltea, and F. Bouchera, Phys. Chem. Chem. Phys. 13, 18539 (2011).

[15]. Z. Y. Zhu, Y. C. Cheng, and U. SchwingenschlÖgl, Phys. Rev. B 85, 235401 (2012).

[16]. D. B. Zax, S. Vega, N. Yellin, and D. Zamir, Chem. Phys. Lett. 138, 105 (1987).

[17]. M. Cardona, Phys. Today 63 (8), 10 (2010).

SUPPLEMENTARY FIGURES

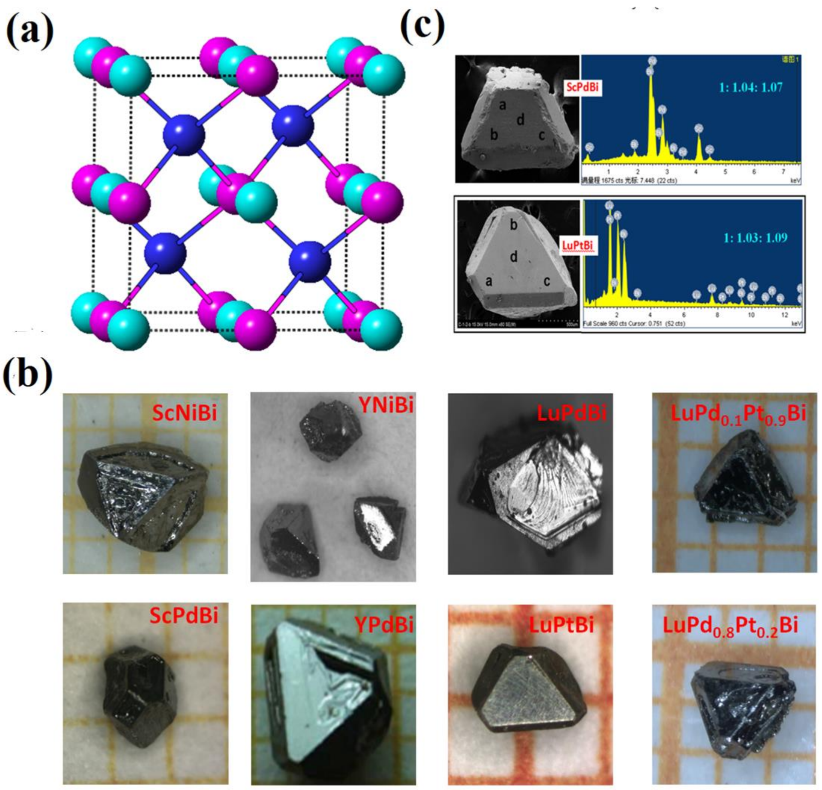

FIG. S1. Synthesis and structures of cubic half-Heusler R-M-Bi single crystals. (a) An illustration of half-Heusler crystal structure (F-43m, S.G.# 216). Transition metal (M, blue); Bi (purple); Rare earth metal (R, cyan). (b) Scanning electron microscopic (SEM) images of six typical single crystals (ScNiBi, ScPdBi, YNiBi,YPdBi, LuPdBi, LuPtBi, $LuPd_{0.1}Pt_{0.9}Bi$ and $LuPd_{0.8}Pt_{0.2}Bi$) after removing excess Bi fluxes; well-developed (111) planes can be observed. (c) The results of SEM-EDX spectrometry with a thin beryllium window system for ScPdBi and LuPtBi, which is used to determine chemical compositions. An EDX analysis was performed at numbered points on the crystal surface and the average R : M : Bi ratio was 1 : 1.04 : 1.07 and 1 : 1.03 : 1.09 for ScPdBi and LuPtBi, respectively, which is nearly equal to the molar ratio 1 : 1 : 1.

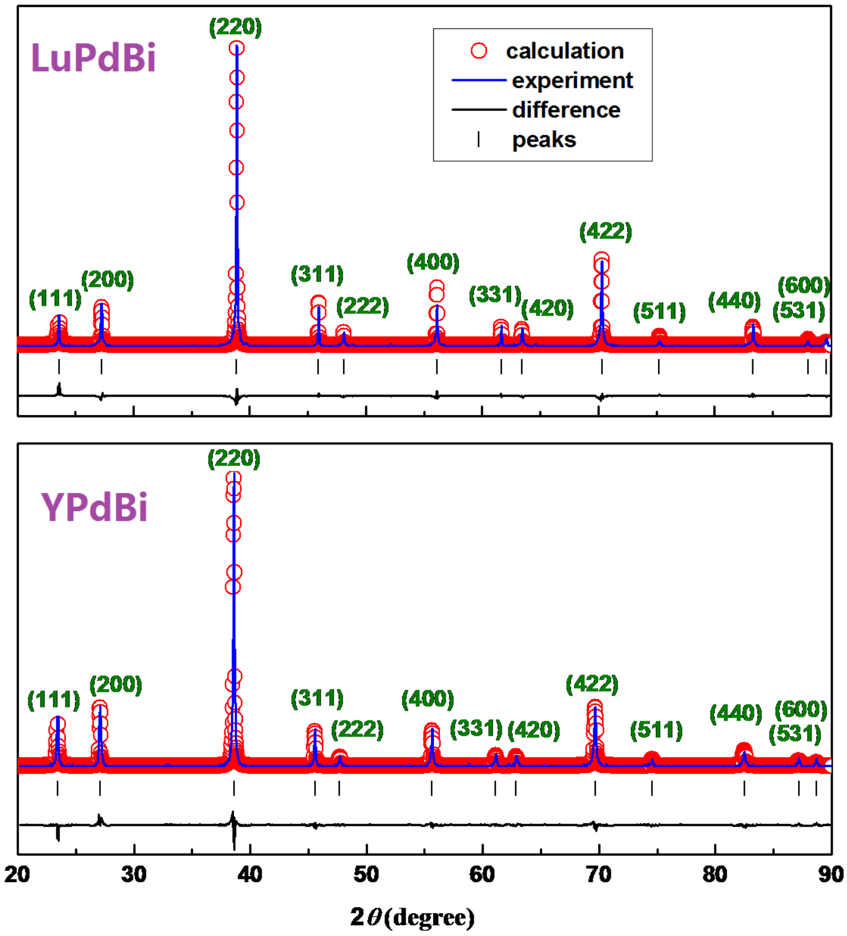

FIG. S2. Synthesis and structures of cubic half-Heusler R-M-Bi single crystals. Observed (red line) powder XRD patterns of crushed LuPdBi and YPdBi single crystals at room temperature and results of structural refinement (green circles). Their XRD reflections are all indexed in the MgAgAs-type structure (space group *F-43m*, 216). Among them, the refined lattice parameters are 6.57 and 6.63 Å for LuPdBi and YPdBi, respectively. These results suggest that our single crystals were of high quality. Only a portion of our results are listed, and we have included some data were taken from our previous work for comparison [5-8].

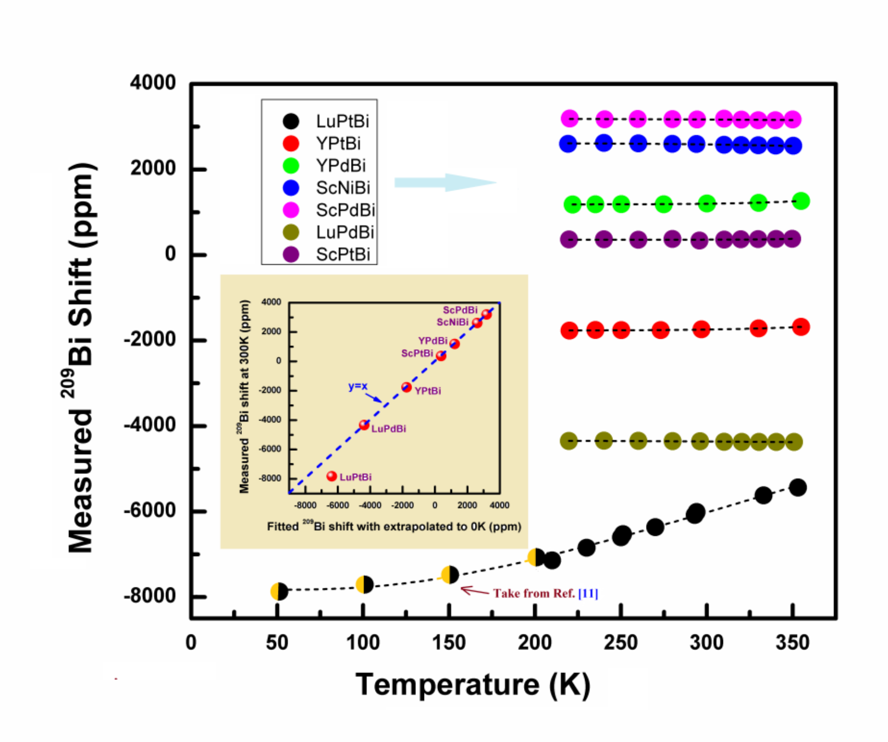

FIG. S3. Temperature dependence of the ^{209}Bi shift of half-Heusler R-M-Bi alloys. Black dashed curves are fitted to Eq. (1). Some of the measured values for LuPtBi (half black/yellow dots) were taken from Ref [11]. The inset shows a comparison of the ^{209}Bi shifts between those measured at 300K and those fitted with an extrapolation 0K. With the exception of LuPtBi, shifts between 300K and 0K were less than 100ppm.

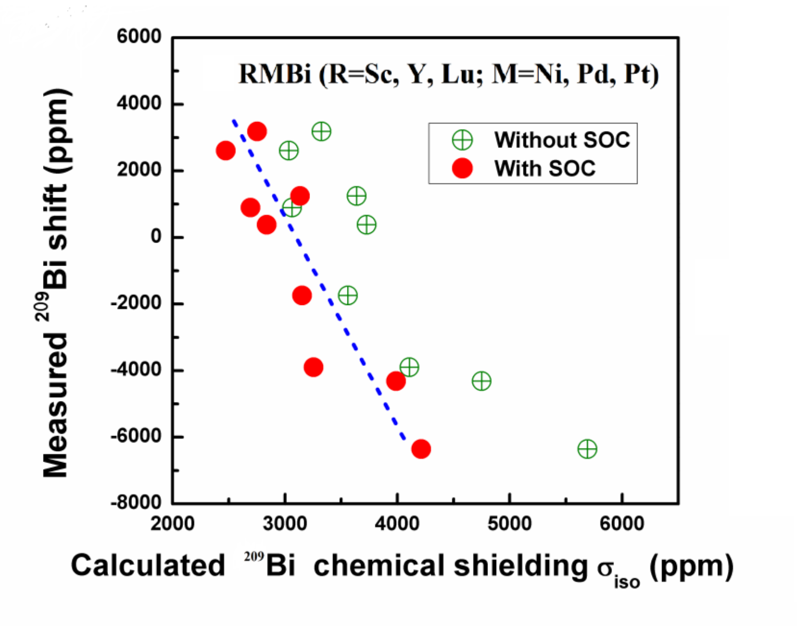

FIG. S4. NMR calculations for half-Heusler R-M-Bi alloys. Calculated ^{209}Bi chemical shielding σ_{iso} values both without (hollow circles with plus) and with SOC (solid circles) for R-M-Bi versus experimentally measured ^{209}Bi shifts are given. This Fig. shows the calculated shifts with respect to SOC follow almost linearly with the measured shifts in contrast to those calculated without SOC. This difference indicates that SOC played a critical role in NMR hyperfine shifts in this alloy system, which is consistent with our other experimental results and the relativistic $p_{1/2}$ shielding via Fermi-contact-like mechanism described in the context of the paper.

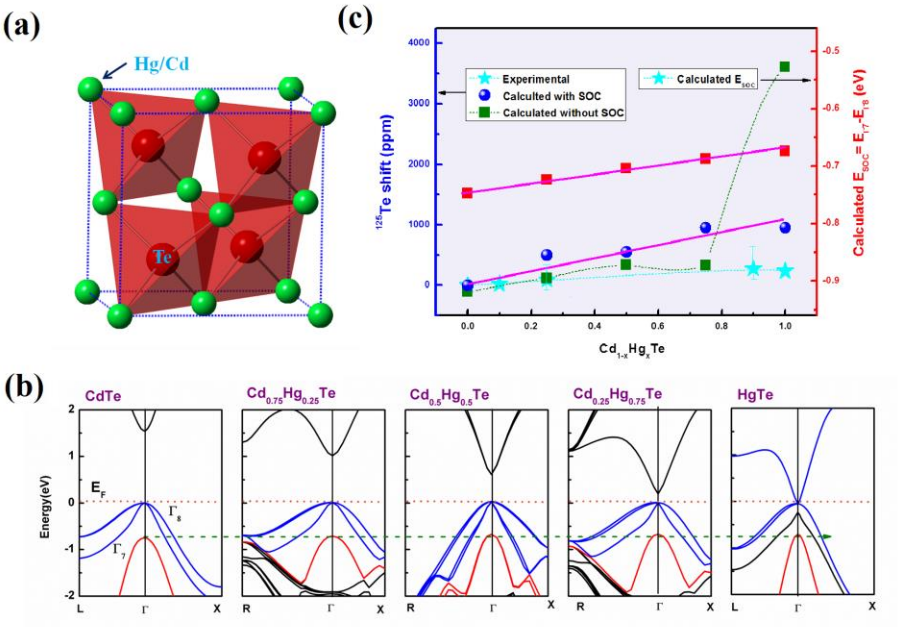

FIG. S5. Discussions for the $Cd_{1-x}Hg_xTe$ (x=0, 0.25, 0.5, 0.75, 1) material system. (a) Crystal structure and (b) electronic band structures under fully relativistic calculation for $Cd_{1-x}Hg_xTe$. (c) The measured (cyan stars) and calculated (blue dots shifts under SOC, olive squares without SOC calculations) NMR ^{125}Te isotropic shifts, and the calculated E_{SOC} (red squares) versus Hg concentrations for the $Cd_{1-x}Hg_xTe$ system. The experimental data were taken from [16]; the calculated values were obtained by setting the ^{125}Te isotropic shifts of CdTe with SOC as zero. The calculated ^{125}Te shifts and E_{SOC} became nearly parallel upon Hg concentration (see pink lines).
